# Pharmacokinetic/pharmacodynamic (PK/PD) evaluation of tulathromycin against *Haemophilus parasuis* in an experimental neutropenic guinea pig model

**DOI:** 10.1371/journal.pone.0209177

**Published:** 2018-12-31

**Authors:** Yongda Zhao, Li-Li Guo, Binghu Fang, Baotao Liu

**Affiliations:** 1 College of Veterinary Medicine, Qingdao Agricultural University, Qingdao, Shandong Province, China; 2 National Risk Assessment Laboratory for Antimicrobial Resistance of Microorganisms in Animals, College of Veterinary Medicine, South China Agricultural University, Guangzhou, Guangdong Province, China; 3 Qingdao Yebio Biological Engineering Co., Ltd, Qingdao, Shandong, China; Universidade Federal do Rio de Janeiro, BRAZIL

## Abstract

The objective of the study was to develop an *ex-vivo* PK/PD model of intramuscular (IM) administration of tulathromycin and to test its efficacy against *Haemophilus parasuis* (*H*. *parasuis*) infection in intraperitoneal-inoculated neutropenic guinea pigs. The pharmacokinetics (PKs) of tulathromycin at doses of 1 and 10 mg/kg in *H*. *parasuis*-infected neutropenic guinea pig were studied by high-performance liquid chromatography-tandem mass spectrometry (HPLC-MS/MS). *In vitro* minimum inhibitory concentration (MIC), minimum bactericidal concentration (MBC), mutant prevention concentration (MPC), post-antibiotic effect (PAE) and dynamic time-kill curve experiments were carried out using *H*. *parasuis* strain 13R. Tulathromycin exhibited concentration-dependent activity and PAE persisted long after administration of the antibiotic. The ratio of the 24-h area under the concentration–time curve (AUC) to MIC in serum (AUC_24h_/MIC_serum_) was recognized as an important PK/PD parameter that positively correlated with the in vitro antibacterial effectiveness of tulathromycin (R^2^ = 0.9961 or R^2^ = 1). For the 1 and 10 mg/kg treatments with tulathromycin, the values of AUC_24h_/MIC for *H*. *parasuis* bacteriostatic action, bactericidal action and virtual bacterial eradication were respectively 22.73, 34.5 and 88.03 h for the 1 mg/kg treatment and respectively 24.94, 30.94 and 49.92 h for the 10 mg/kg treatment. In addition, we demonstrated that doses of 7.2–8.0 mg/kg of tulathromycin resulted in high eradication rates (99.99%). Using a previously published conversion factor of 0.296, we were able to estimate an approximate dose, 2.1–2.4 mg/kg, that should also obtain high eradication rates in the target animal, pigs. This study can help optimize tulathromycin efficacy against *H*. *parasuis* infections in swine farming.

## Introduction

Tulathromycin is a semi-synthetic macrolide approved solely for veterinary use by the US Food and Drug Administration (FDA) and European Medicines Agency (EMA). Because of its potent activity, tulathromycin is commonly used to treat bovine respiratory (BRD) and swine respiratory diseases (SRD) [1, 2]. *H*. *parasuis*, a Gram-negative bacterium, is included in the list of indications that has been approved by both the FDA and EMA because it is a significantly harmful pathogen in contemporary swine production systems worldwide [2–4].

Antimicrobial drugs are the most effective and frequently used treatment in veterinary medicine [5]. However, antibiotic use has an unwanted side effect of selective pressure on the target bacterial species in a treated animal and its environment [5]. Thus, the risk of selection for antibiotic resistance and amplification of bacteria is as important to address as clinical efficacy [6–8] in antimicrobial drug development and application. The risks warrant the need to optimize antimicrobial drug dosage. However, dosing regimens of most antibacterial drugs in the world market have been based on the PKs and MICs of the drugs against pathogens [5], which do not reflect the dynamic interactions between drugs, hosts and pathogenic microbes. Furthermore, the doses labeled on bottles of antibiotics have not been suitable for veterinarian use. Because more robust information can be provided by using PK/PD modeling approaches, their use in drug development for human and veterinary medicine has been increasing [9–11]. Moreover, regulatory agencies (e.g. EMA and FDA) now recommend using PK/PD modeling to establish more ideal dosage schedules for both old and new drugs currently being used in veterinary practice to eradicate bacteria [5, 12, 13]. To the best of our knowledge, macrolide antibiotics are time-dependent agents with prolonged post-antibiotic effects [14]. Few studies have reported on macrolide antibiotics in PK/PD modeling, thus, we applied PK/PD modeling in an *H*. *parasuis*-infected, neutropenic guinea pig model.

This study has two objectives: (i) to examine the PKs of tulathromycin in infected neutropenic guinea pigs using two dose levels and determine optimal *in vitro* PD parameters and *ex-vivo* PD characteristics of tulathromycin, both in cation-adjusted Mueller-Hinton broth (CAMHB) and serum; and (ii) to integrate the parameters and characteristics in a PK/PD model of tulathromycin treatment of systemic *H*. *parasuis* infection.

## Materials and methods

### Animals and ethic statement

A total of 266 FMMU albino guinea pigs (three-week old, 240–250 g) were purchased from the Laboratory Animal Center of Southern Medical University in Guangzhou, China (License number: SCXK (YUE) 2011–2015). Guinea pigs were housed in the Laboratory Animal Center of South China Agriculture University, where all guinea pigs had free access to antibacterial-free food and water. In the Laboratory Animal Center, guinea pigs were raised in cages, four per cage. The guinea pigs were maintained on a 12-h/12-h light/dark cycle, and the room temperature and relative humidity were maintained at 25±2°C and 55%±5%, respectively. This study was carried out in strict accordance with the recommendations in the Guide for the Care and Use of experimental animals. The protocol was approved by the South China Agriculture University Animal Ethics Committee (Protocol Number: 2014–025). People who participated in the experiment, had been trained according to the recommendations in the Guide for the Care and Use of experimental animals before the experiment. All surgery was performed under sodium pentobarbital anesthesia, and all efforts were made to minimize suffering.

### Experimental design

After waiting three days after receipt of the animals, each guinea pig was rendered neutropenic by intraperitoneal injection (IP) of 100 mg/kg of cyclophosphamide (TCI (Shanghai) Development Co., Ltd, Shanghai, China) at a frequency of one injection per day for three days. Blood was drawn from the heart and leukocytes were counted with an automatic blood cell analyzer (Mindray BC-2800Vet, Shenzhen, China). An *H*. *parasuis* standard strain of serotype 13 (13R) was used to infect the neutropenic guinea pigs via intraperitoneal injection, specifically, a single inoculum of a 0.2 mL aliquot solution containing approximately 10^9^ CFU of the *H*. *parasuis* strain, which was a 95% infective dose (ID_95_) that was previously determined in pilot studies. Ten guinea pigs were used to detect blood and lung tissue bacterial loads in a 72 h-period, and 256 guinea pigs were used for PKs of tulathromycin in a 168 h-period. To obtain antimicrobial PK data, infected neutropenic guinea pigs were given single IM doses at two levels, 1 or 10 mg/kg, via tulathromycin Injection (Draxxin®) (Zoetis, New York, USA).

### Sample collection

To quantify pathogen load in the *H*. *parasuis* intraperitoneal infection model, blood samples were collected from the heart before euthanasia and viable counts (CFU/mL) were detected immediately, and lung tissues were removed at sacrifice by using an overdose of sodium pentobarbital on each animal at about 2 h, when typical symptoms of the respiratory tract were observed, which included appearing depressed, crowding together, closing eyes, drooping, coughing, and abdominal breathing, and 72 post-challenge. No guinea pigs died in a 72h-period. Viable counts (CFU/g) of bacteria in lung tissues were detected within one hours of tissue removal. Five guinea pigs were sampled at each time point.

To obtain pharmacokinetic data, aliquots of 2 mL of blood samples were collected from the heart at 0.083, 0.167, 0.25, 0.5, 1, 2, 4, 8, 12, 24, 48, 72, 96, 120, 144, and 168 h after drug administration. Eight guinea pigs were sampled at each time point.

### Analysis method

#### Chemicals and reagents

Tulathromycin (99.85%) was supplied by Shandong Lukang Pharmaceutical Co., Ltd (Shandong, China). Roxithromycin was purchased from National Institutes for Food and Drug Control (Beijing, China) and used as an internal standard. HPLC grade methanol, acetonitrile and formic acid were used for drug analysis. Ultrapure water was provided by a Milli-Q ultrapure water purification system (Milli-Q Millipore Corp.).

#### Viable count

Lung tissue (0.5 g) was added into 0.5 mL of phosphate-buffered saline (PBS), and centrifuged at 7200 rpm for 15 seconds by a Precellys Evolution Super Homogenizer (Bertin Technologies, France). Aliquots of 0.1 mL supernatant were used in 10-fold serial dilutions to obtain viable count data (CFU/mL). We removed 20 μL from each dilution to spread on tryptic soy agar (TSA) plates containing 10 μg/mL nicotinamide adenine dinucleotide (NAD, Sigma, Inc., USA) and 5% bovine serum (Gibco®, New Zealand). The colonies were counted (CFU) after incubation for 36–48 h at 37°C. Blood samples were collected from the heart before euthanasia and viable count data was collected. The lowest detectable count was 10 CFU/mL. All samples were performed in triplicate.

#### Drug analysis

Serum was obtained by centrifuging blood samples at 4,000 rpm for 10 min and immediately stored at -20°C until analysis (within one month of sampling). Tulathromycin concentrations in serum and lung tissue were determined via HPLC-MS/MS method as described previously [15, 16]. The free fraction (*fu*) of tulathromycin in guinea pig serum was determined in triplicate using Amicon Centrifree Micropartition devices (Millipore, Bedford, MA, USA) with a 10000 Nominal Molecular Weight Limit according to the manufacturer’s instructions. To measure PK parameters of guinea pig serum, 0.5 mL was added into an ultrafiltration device and then centrifuged at 12000 g for 45 min at 25°C. Measurements for PK parameters were taken using samples obtained at 0.5, 2, 8, 24 and 48 h. The concentration of tulathromycin was determined by HPLC-MS/MS.

#### Pharmacokinetics analysis

The PK parameters of tulathromycin in serum were analyzed by WinNonlin software (version 5.2; Pharsight, CA, USA). A WinNonlin model 200 was used for non-compartmental analysis of the concentration-time data. PK parameters were expressed as mean values ± standard deviations (SD).

#### *In vitro* and *ex vivo* susceptibility studies and PAE

*H*. *parasuis* was cultured in as a previous report[17]. A total of 94 strains of *H*. *parasuis* strains isolated from swine between 2014 and 2016 were used in this study. The MIC and MBC of tulathromycin against *H*. *parasuis* were determined in both CAMHB cultures and serum by the micro dilution method according to protocols acquired from the Clinical and Laboratory Standards Institute [18]. The concentrations ranged from 0.015 to 8 μg/mL. Tests were conducted in triplicate and included growth controls (*H*. *parasuis* in the media only), and germ-free controls (blank media only). The MIC value is defined as the lowest tulathromycin concentration exhibiting no visible growth of *H*. *parasuis* when bacteria were cultured at 37°C for 24 h. The MBC is defined as the lowest drug concentration which resulted in a 99.9% reduction of bacterial density when bacteria were cultured at 37°C for 24 h.

The measure of MPC of tulathromycin against *H*. *parasuis* 13R was determined by the agar method [19]. A final concentration of ∼3 × 10^10^ CFU/mL was used in determining MPC. Samples (100 μL) were plated onto TSA agar containing various concentrations of tulathromycin obtained by successive two-fold dilutions. The MPC was measured at MIC levels of 1, 2, 4, 8, 16, 32 and 64. All plates were incubated at 37°C for 72 h and then examined for growth. The MPC represents the lowest antibiotic concentration where no bacterial growth was observed while under anaerobic conditions. Samples were performed in triplicate.

The PAE of tulathromycin was estimated by the removal of drug method [20]. *H*. *parasuis* 13R was incubated with 1, 2 and 4 MIC of tulathromycin. After incubating for 1 or 2 h, he drug was removed by dilution 1000 times with fresh medium. The viable counts of *H*. *parasuis* were determined at 1, 2, 4, 6, 8, 10 and 12 h. The PAE was calculated as follows [20]: PAE = T-C;, where T and C represent the time periods required for viable counts of bacteria to increase by 1-log10 CFU in the drug removal phase for the treatment and the untreated control groups, respectively.

#### *In vitro* and *ex vivo* time-killing curves

*In vitro* time-killing curves of tulathromycin against *H*. *parasuis* were obtained after determination of MIC and MBC values. Serial concentrations of tulathromycin were prepared in CAMHB and guinea pig serum ranging from 0.125 to 32 MIC before bacterial inoculation (10^6^ CFU/mL). Growth and sterile controls were performed at the same time. The total volume for each concentration was 4 mL. The cultures were incubated at 37°C for 24 h, and the viable counts of bacteria were determined at 0, 2, 4, 6, 8, 10, 12 and 24 h of incubation time. The limit of detection was 10 CFU/mL. All samples were performed in triplicate.

All serum samples obtained from guinea pigs (0–168 h) after IM injection of tulathromycin were used to establish e*x-vivo* time-killing curves. A control serum was prepared from samples collected from the same guinea pig before administration. All serum samples were pre-filtered through a 0.22 μm membrane to eliminate any pathogens. In order to reduce potential variability in growth rates of bacteria between the *in vitro* and *ex-vivo* experiments, the same volume of 4 mL as CAMHB samples and same incubation time of 24 h was used as described above. We averaged the values measured from the sera of each group of eight guinea pigs per time point to plot e*x vivo* time-killing curves. The viable counts of bacteria were determined as described above.

#### PK/PD modeling and data analysis

The surrogate markers of antibacterial effectiveness of tulathromycin, AUC_168h_/MIC, T>MIC, and C_max_/MIC, were calculated using *in vitro* MIC values and PK parameters obtained from IM administrations of tulathromycin in serum samples. The effectiveness of tulathromycin was expressed as changes in log_10_ CFU after 24 h of incubation of serum samples used to establish *ex-vivo* time-killing curves. The *in vitro* PK/PD relationship of tulathromycin was described using a sigmoid inhibitory *E*_max_ model with the WinNonlin software (Version 5.2; Pharsight, CA, USA) and the equation is as follows:

$$E=E_{max}-(E_{max}-E_{0})\times \frac{C_{e}^{N}}{{EC}_{50}^{N}+C_{e}^{N}}$$

where *E* is the antibacterial effect measured as the reduction in log_10_ CFU/mL after administration of tulathromycin compared to the log_10_ CFU/mL in the untreated control group; *E*_*max*_ is the reduction in log_10_ CFU/mL for the untreated control guinea pigs; *E*_*0*_ is the maximum reduction after administration and represents the maximum antibacterial effect; *Ce* is the AUC_0-24h_/MIC parameter; *EC*_*50*_ is the AUC_0-24h_/MIC_serum_ value required to achieve 50% of the maximal antibacterial effect; and *N* is the Hill coefficient that describes the steepness of the AUC_0-24h_/MIC_serum_ and effect curve.

#### Dose regimen prediction

In order to determine an optimal dose regimen to apply for treatment of *H*. *Parasuis* in veterinary medicine, the dose required for a given level of antibacterial activity is predicted by the equation [21]:

$$Dose=\frac{{Cl}_{for\;10\;days}\times factor\times {MIC}_{90}}{fu\times F}$$

where Dose is the optimal dose to produce and sustain antibacterial activity for 10 days (mg/kg/10-day); Cl is the serum clearance; factor is the dimensionless numerical value of AUC/MIC_serum_; MIC_90_ is the 90th percentile of the MIC distribution; *fu* is the free drug fraction; and *F* is the bioavailability of tulathromycin.

## Results

### Pharmacokinetics of tulathromycin

**Fig 1:**
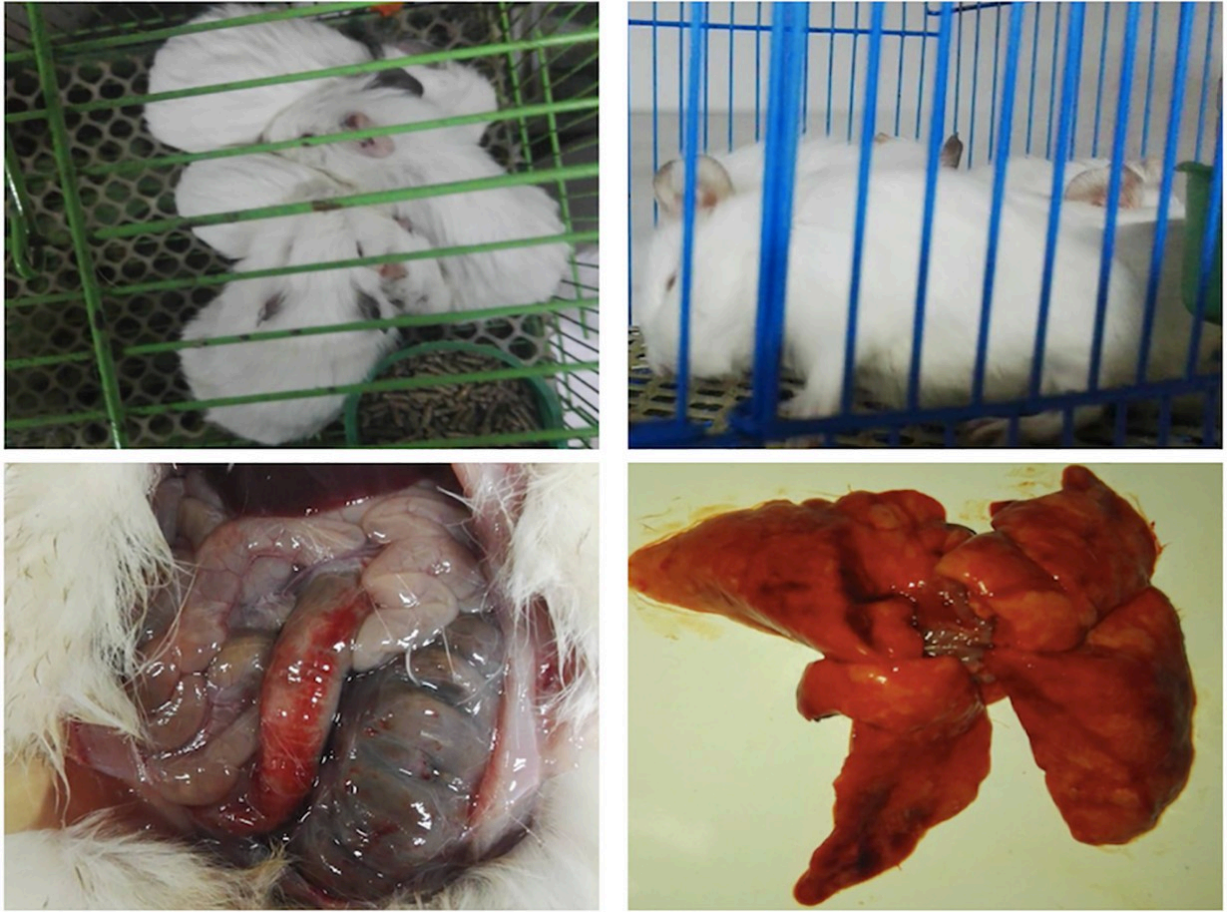
Clinical symptoms after *H*. *parasuis*-infection in guinea pigs. Top left panel, guinea pigs group together and exhibit immunocompromised; top right panel, a guinea pig’s head droops with closed eyes; bottom left panel, celiac effusion and intestinal mucosal hemorrhage; and bottom right panel, liver swelling, necrosis and hemorrhage.

Animals were severely granulocytopenic, and clinical symptoms and bacteriological examinations verified that neutropenic guinea pigs were successfully infected with *H*. *parasuis* (Table 1, Fig 1).

**Table 1:** Absolute leukocyte count and mean *H*. *parasuis* load in serum or lung tissue post-infection by *H*. *parasuis* (Mean ± SD, n = 5).

	*H*. *parasuis*-infection stage	
	2 h after infection	3 d after infection
**Absolute leukocyte count (mm**^**3**^**)**	<1000	<1000
**Mean *H*. *parasuis* load in serum (CFU/mL)**	6.03 ± 1.12	5.77± 0.88
**Mean *H*. *parasuis* load in lung (CFU/g)**	6.16 ± 0.78	5.70 ± 0.79

**Fig 2:**
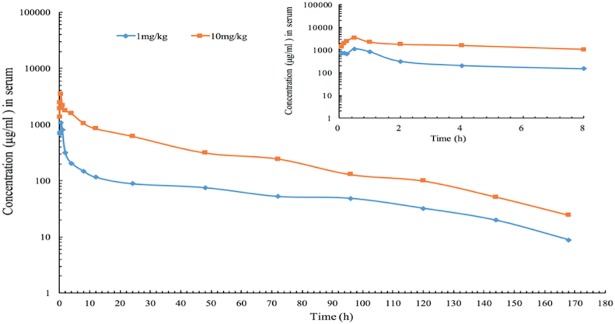
The concentration-time curve of tulathromycin in guinea pig serum after a single dose intramuscular administration at 1 or 10 mg/kg in a H. parasuis infection model (inset depicts the serum concentrations in the first 8-h post-administration) (n = 8/time point).

The PK parameters of the two dose levels are shown in Table 2 and the serum concentration-time profiles are illustrated in Fig 2. After IM administration, tulathromycin was rapidly absorbed and its concentrations peaked at 0.5 h at mean values (C_max_) of 1079.5 and 3486.25 ng/mL for the 1 mg/kg and 10 mg/kg doses, respectively. The mean elimination half-lives of the two respective dose levels were 25.8 and 26.9 h, which indicate slow elimination rates. The mean area under the concentration-time curve (AUC_0−168h_) were 11019.6 and 57182.5 ng.h/mL, respectively. Mean body clearance rates (Cl) were 88.1 and 172.0 mL/kg/h, respectively. Additionally, the free fraction (*fu*) of tulathromycin in guinea pig serum was 0.56–0.74.

**Table 2:** Pharmacokinetic parameters of tulathromycin in serum following a single dose intramuscular administration at 1 or 10 mg/kg in *H*. *parasuis*-infected guinea pigs (Mean ± SD, n = 128/group).

Parameter*	Unit	1 mg/kg	10 mg/kg
**T**_**max**_	**h**	0.5 ± 0.1	0.5 ± 0.2
**C**_**max**_	**ng/mL**	1079.5 ± 270.8	3486.25 ± 453.5
**T**_**1/2**_	**h**	25.8	26.9
**Cl**	**mL/kg/h**	88.1	172.0
**AUC**_**0−168h**_	**ng•h/mL**	11019.6	57182.5

* T_max_, time of maximum concentration; C_max_, maximum concentration; T_1/2_, elimination half-life; Cl, body clearance; AUC_0-168h,_ 168 h area under concentration-time curve.

### MICs, MBCs, MPC and PAE of tulathromycin activity against *H*. *parasuis*

The MIC and MBC of tulathromycin against 94 strains of *H*. *parasuis* in CAMHB and serum are shown in Table 3. The ranges of MIC in CAMHB and serum were 0.06 to 8 μg/mL and 0.0075 to 0.25 μg/mL, respectively. The ranges of MBC in CAMHB and serum were 0.125 to 8 μg/mL and 0.0075 to 0.25 μg/mL, respectively. The MBC/MIC ratios ranged from 8.33 to 33.33 in CAMHB and 4 to 33.33 in serum. The values of MIC_90_ of 94 strains of *H*. *parasuis* strains isolated from pigs were 0.5 μg/mL in CAMHB culture and 0.06 μg/mL in guinea pig serum. In addition, the MICs of *H*. *parasuis* 13R in CAMHB and in guinea pig serum were 0.5 and 0.03 μg/mL, respectively. The MBCs of *H*. *parasuis*13R in CAMHB and in guinea pig serum were 1 and 0.06 μg/mL, respectively. Additionally, the MPC of tulathromycin against *H*. *parasuis* 13R in TSA was 2.048 μg/mL.

**Table 3:** MIC and MBC of tulathromycin against *H*. *parasuis* (Mean ± SD, n = 94).

Test matrix	MIC (μg/mL)	MBC (μg/mL)	MBC/MIC ratio
**CAMHB**	0.06–8	0.125–8	1–4
**Guinea pig serum**	0.0075–0.25	0.0075–0.5	1–4.17
**CAMHB/Serum ratio**	8.33–33.33	4–33.33	/

PAE of different concentrations of tulathromycin (1x, 2x, or 4x MIC) and exposure times (1 or 2 h) are shown in Table 4. *H*. *parasuis* 13R was sensitive to tulathromycin. After 1 h of exposure at a concentration of 1, 2 or 4 MIC, PAEs were 0.38, 0.77 and 1.24 h, respectively. While after the 2-h exposure, the PAEs for the same three concentrations were 1.10 h, 2.85 h and 8.06 h, respectively.

**Table 4:** Post antibiotic effect (PAE) after 1 and 2 h on *H*. *parasuis* strain 13R.

Antibacterial concentration	PAE after 1h (h)	PAE after 2h (h)
**1MIC**	0.38	1.10
**2MIC**	0.77	2.85
**4MIC**	1.24	8.06

### *In vitro* and *ex vivo* antimicrobial activities of tulathromycin

**Fig 3:**
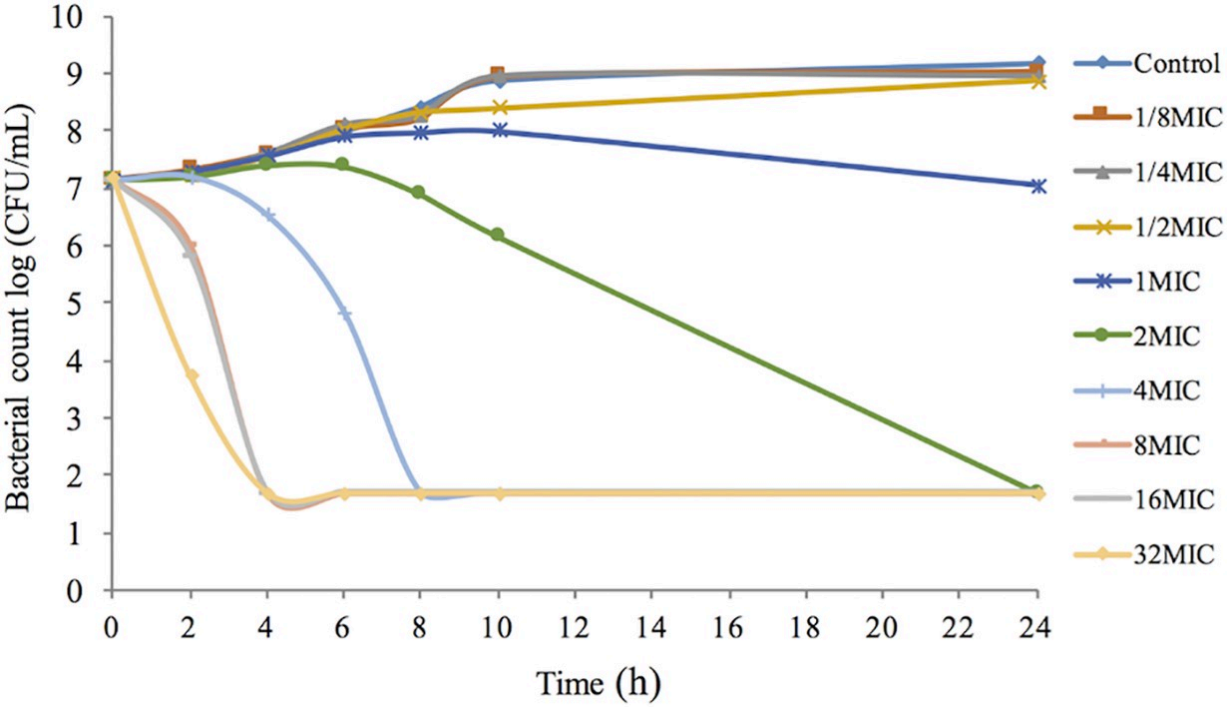
*In vitro* time-kill curves of tulathromycin against *H*. *parasuis* 13R in CAMHB medium (MIC_CAMHB_ = 0.5 μg/mL).

**Fig 4:**
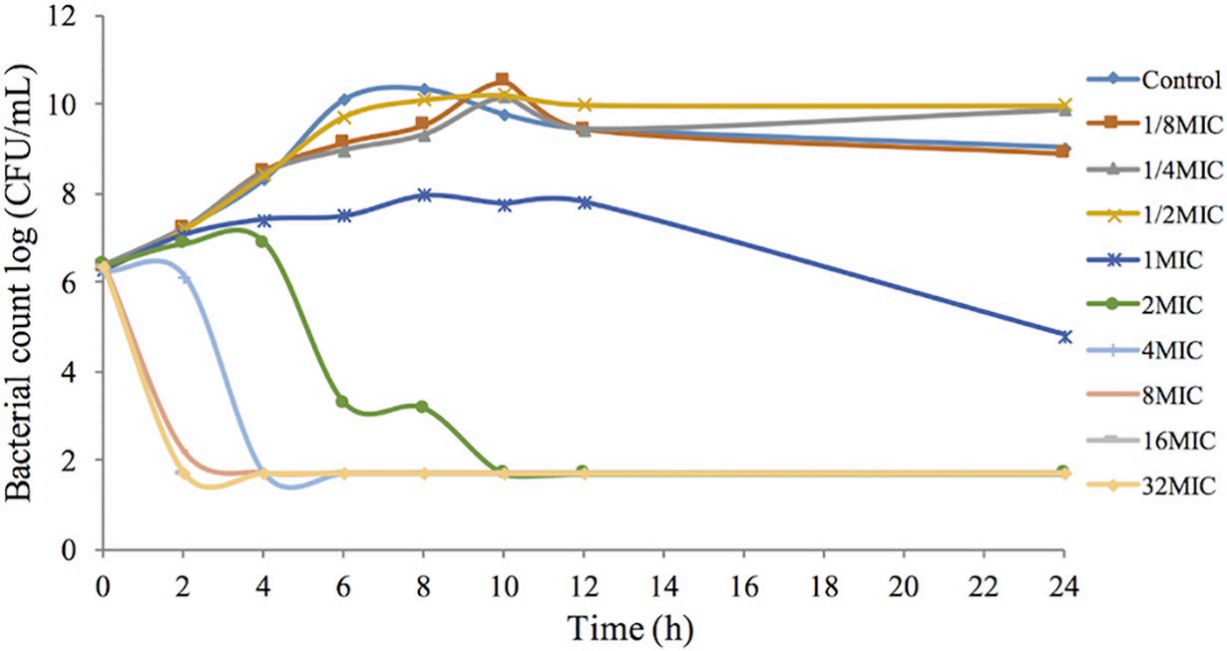
*Ex-vivo* antibacterial activity of tulathromycin against *H*. *parasuis* 13R in blank guinea pig serum samples with added tulathromycin (MIC_serum_ = 0.03 μg/mL).

*In vitro* time-kill curves obtained for antimicrobial activity against *H*. *parasuis* 13R for different concentrations of tulathromycin in the range of 0.125–32x of the MIC are shown in Fig 3. The concentrations less than the MIC of tulathromycin exhibited similar levels of antimicrobial activity against *H*. *parasuis*. However, when tulathromycin concentrations were higher than the MIC, the bacteriocidal activity gradually improved due to the increase in drug concentration, which supports the notion that the concentration-dependent response is a characteristic of tulathromycin activity. The time-kill curves of tulathromycin against *H*. *parasuis* 13R in the range of 0.125–32x of the MIC were also obtained from blank guinea pig serum and infected guinea pig serum samples, both of which were treated with tulathromycin (Fig 4). Similar antimicrobial activity of tulathromycin against *H*. *parasuis* was observed in guinea pig serum. The bactericidal activity increased with increasing concentration of tulathromycin up to 2 MIC. Additionally, when bacterial counts were reduced by roughly 4-log_10_CFU/mL with increasing concentration of tulathromycin, it occurred at a shorter time period of 2–4 h.

**Fig 5:**
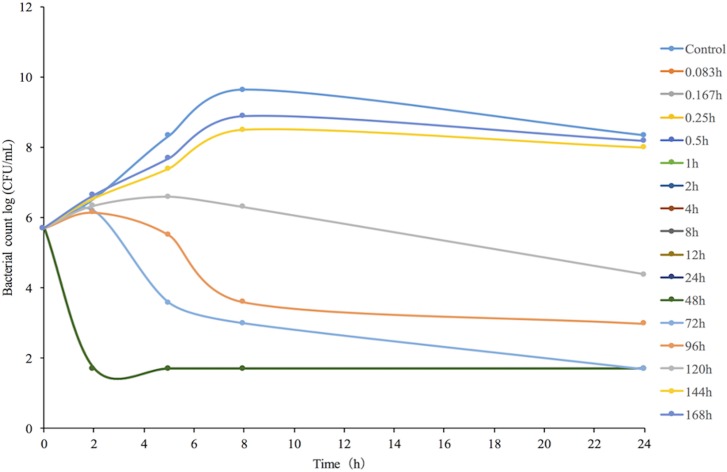
*Ex-vivo* antibacterial activity of tulathromycin in serum of guinea pigs against *H*. *parasuis* after IM administration (1 mg/kg.b.w., n = 8/per time point).

**Fig 6:**
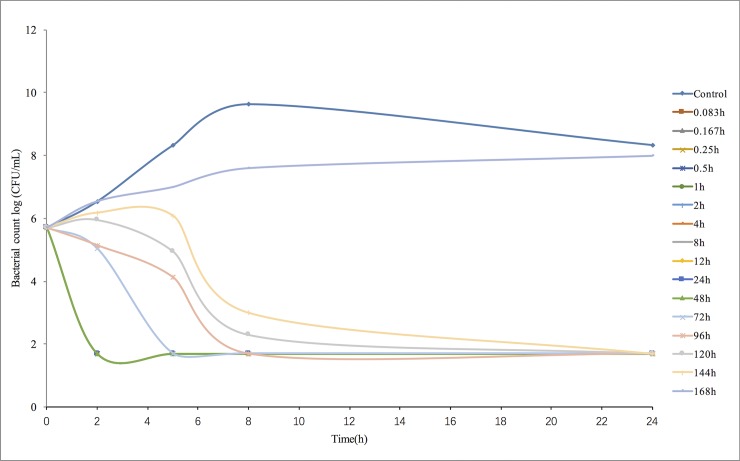
*Ex-vivo* antibacterial activity of tulathromycin in serum of guinea pigs against *H*. *parasuis* after IM administration (10 mg/kg.b.w., n = 8/per time point).

Data from guinea pig serum samples that had IM administrations with one of the two dose levels of tulathromycin and their blood collected at different time points (0–168 h) were used to determine an *ex-vivo* killing rate. In the 1 mg/kg treatment (Fig 5), no bactericidal activity was observed for serum collected at 144 or 168 h. However, in the 10 mg/kg treatment and within the range of concentrations of 0.31 to 3.45 μg/mL, the number of bacteria decreased slightly after 0.083–48 h (Fig 6). Additionally, no bactericidal activity was measured in serum collected at 168 h.

### PK/PD integration and dose regimen prediction

**Fig 7:**
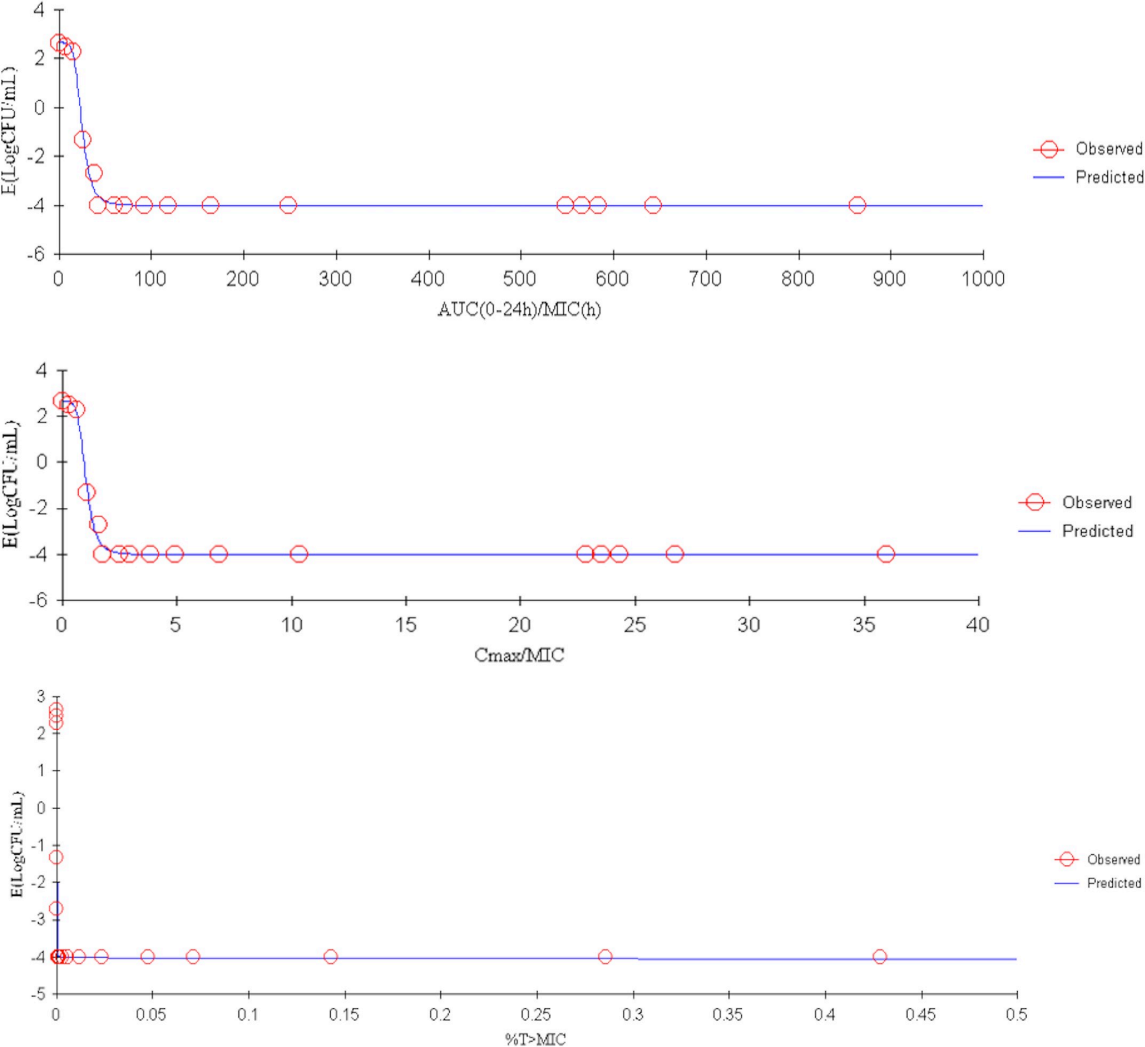
Sigmoid *E*_*max*_ relationship between anti-*Haemophilus parasuis* effect (*E*, log_10_ CFU/serum) and *ex vivo* AUC_24h_/MIC ratio in the serum of guinea pigs based on a single dose of 1 mg/kg of tulathromycin.

**Fig 8:**
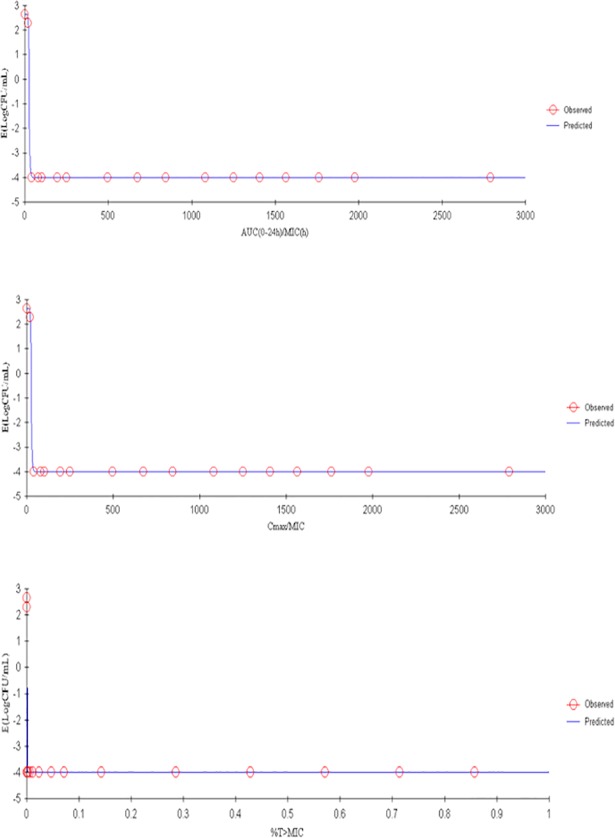
Sigmoid *E*_*max*_ relationship between anti-*Haemophilus parasuis* effect (*E*, log_10_ CFU/serum) and *ex vivo* AUC_24h_/MIC ratio in the serum of guinea pigs based on a single dose of 10 mg/kg of tulathromycin.

The ratios of PK/PD parameters C_max_/MIC_serum_, AUC_168h_/MIC_serum_, C_max_/MBC_serum_, AUC_168h_/MBC_serum_, C_max_/MPC_CAMHB_, AUC_168h_/MPC_CAMHB_ of tulathromycin activity against *H*. *parasuis* are shown in Table 5. According to these ratios and the sigmoid *E*_*max*_ model, the PK/PD indices AUC_0-24h_/MIC_serum_ and C_max_/MIC were the best predictors of tulathromycin efficacy against *H*. *parasuis*. AUC_0-24h_/MIC, %T>MIC, C_max_/MIC and the degree of antibacterial effect are displayed in Fig 7 and Fig 8.

**Table 5:** Ratios of PK/PD data sampled from guinea pigs after IM administration of tulathromycin at 1 or 10 mg/kg (Mean ± SD).

Parameters	Unit	Values	
		1 mg/kg	10 mg/kg
**C**_**max**_**/MIC**_**serum**_	**-**	36.0 ± 9.0	116.3 ± 15.1
**AUC**_**168h**_**/MIC**_**serum**_	**h**	367.3 ± 79.4	1906.0 ± 435.1
**C**_**max**_**/MBC**_**serum**_	**-**	18.0 ± 4.5	58.2 ± 7.6
**AUC**_**168h**_**/MBC**_**serum**_	**h**	183.7 ± 39.7	953.0 ± 217.6
**C**_**max**_**/MPC**_**TSA**_	**-**	0.5 ± 0.1	1.7 ± 0.2
**AUC**_**168h**_**/MPC**_**TSA**_	**h**	5.4 ± 1.2	27.9 ± 6.4

We quantified PK/PD parameters and the antibacterial effects of tulathromycin and presented them in Table 6. We characterized tulathromycin efficacy by three levels: (1) bacteriostatic activity, where there was no change from the initial inoculum count of bacteria after a 24-h incubation period (*E* = 0); (2) bactericidal action, where 99.9% of the original inoculum count was reduced after a 24-h incubation period (*E* = -3); and (3) virtual eradication, where 99.99% of the original inoculum count was reduced after a 24-h incubation period (*E* = -4). For both dose treatments, 1 and 10 mg/kg, the AUC_0−24_/MIC ratios of serum samples for each of the three levels of efficacy were, respectively, 22.73, 34.52 and 88.03 for the lower dose treatment and, respectively, 24.94, 30.94 and 49.92 for the higher dose treatment.

**Table 6:** Results from a PK/PD model of *ex-vivo* data sampled from guinea pigs administered with tulathromycin of one of two dose levels.

Parameters	1 mg/kg	10 mg/kg
***E***_***0***_ * **(log**_**10**_ **CFU/mL)**	-4.01	-4.01
***E***_max_* **(log**_**10**_ **CFU/mL)**	2.65	2.65
***EC***_50_*	24.66	26.0
Slope (N*)	5.11	10.0
**R**^**2**^	0.9961	1
**AUC**_**0-24h**_**/MIC**_**serum**_ **for bacteriostatic activity**	22.73	24.94
**AUC**_**0-24h**_**/MIC**_**serum**_ **for bactericidal action**	34.52	30.94
**AUC**_**0-24h**_**/MIC**_**serum**_ **for and virtual bactericidal eradication**	88.03	49.92

*E_*0*_ is the change in log_10_ CFU/mL in the control sample (absence of tulathromycin); *E*_*max*_ is the difference in effect between the greatest amount of growth (observed in the growth control, *E*_*0*_) and the greatest amount of mortality. *EC*_*50*_ is the AUC_0-24h_/MIC_serum_ value at which a 50% reduction in bacterial counts was observed. N is the Hill coefficient that describes the steepness of the AUC_0-24h_/MIC-effect curve.

After testing two dose levels, 1 mg/kg and 10 mg/kg, by a single intravenous administration in infected guinea pigs, we were able to predict the dose of tulathromycin necessary to virtually eradicate an *H*. *parasuis* infection in guinea pig by using the values of AUC_24h_/MIC from the PK/PD model and of *fu* and MIC_90serum_ obtained in our study. We estimated that for the 1 mg/kg and 10 mg/kg treatments, a dose range between 7.2 and 8.0 mg/kg would achieve the virtual eradication of *H*. *parasuis* over a ten-day period.

## Discussion

*H*. *parasuis* can cause systemic infection in pigs, including anorexia, depression tremors, posterior paresis or lateral recumbency, and incoordination [22–24]. In the present study, we established a neutropenic guinea pig model where we successfully infected with *H*. *parasuis* by intraperitoneal injection, to study the efficacy of tulathromycin and to eliminate the influence of individual differences due to immunity among guinea pigs. Evaluation of the *H*. *parasuis* infection model relied first on clinical symptoms, and then on bacteriological assays. In the present study, clinical symptoms and bacteriological examinations were observed and detected in infected animals from 2 h post-infection, which indicated guinea pigs infected with *H*. *parasuis* produce clinical and dissection symptoms similar to infected pigs, and the guinea pig model can replace the target animal to study *H*. *parasuis*.

In this study, after single IM doses of 1 or 10 mg/kg of tulathromycin, the mean T_1/2_ were 25.8 h and 26.9 h, respectively. These results are similar to results of previously published articles on laboratory animals other than guinea pigs such as mice [25] and rabbit [26]. However, our mean T_1/2_ were shorter than the results of studies that used target animals, such as pig, cattle, goats, and horses [27–32]. Other studies’ PK parameters were similar to our pharmacokinetic results, such as the rapid rate of absorption, wide extent in systemic availability, and extensive tissue distribution [25–29, 31]. Additionally, the free fraction (*fu*) of tulathromycin in guinea pig serum (0.56–0.74) was similar in range to a previous report (0.53–0.68) using swine serum [13].

Between 2000 and 2002, studies in the U.S. and Canada reported MICs of tulathromycin against *H*. *parasuis* from 0.25 to >64 μg/mL and an average MIC_90_ of 2 μg/mL [13], and between 2008 and 2011, studies in the Czech Republic reported MICs from 0.5 to 64 μg/mL and an average MIC_90_ of 8 μg/mL [33]. In the present study, the MIC of tulathromycin against *H*. *parasuis* was from 0.06 to 8 μg/mL for 94 bacterial strains and MIC_90_ was 0.5 μg/mL in the CAMHB, whereas in serum, the MIC was 0.0075 to 0.25 μg/mL and the MIC_90_ was 0.06 μg/mL. The CAMHB/serum ratio of MIC ranged from 8.33–33.33, which indicated that the MIC measured in serum was significantly lower than the MIC measured in broth. Similar results have been reported in several previous studies [34–36]. The MBC measured in this study showed similar CAMHB/serum ratios to the MIC. This finding and findings of other researchers indicate that tulathromycin may have a strong effect on serum, and thus, the MIC in serum is likely more applicable than in CAMHB to establish a rational dosing regimen in *in vivo* or *in vitro* antimicrobial activity studies [34–38].

*Ex-vivo* PK/PD of tulathromycin has been studied recently, against *Pasteurella multocida* [35] and *Streptocossus suis* [34] in pigs, and against *Mannheimia haemolytica* and *Pasteurella multocida* [36] in cattle. To the best our knowledge, most macrolide drugs are time-dependent antibacterial agents, where the efficacy of these drugs depends on the time period at which the drug concentration is above the MIC. For time-dependent antibacterial agents, T>MIC and AUC/MIC were the best indices to use in PK/PD modeling [5, 39]. Recent studies have reported different bactericidal activity of tulathromycin against different bacteria, for example, a time-dependent killing pattern against *Streptococcus suis* [34] and an *in vitro* concentration-dependent bactericidal activity against *Haemophilus somnus* [40, 41] and *Pasteurella multocida* [35]. In the present study, the evaluation of activity of tulathromycin *in vitro* showed that tulathromycin had concentration-dependent bactericidal activity against *H*. *parasuis*, which supports a previous report that also concluded a positive relationship between drug concentration and bacterial killing [5].

For concentration-dependent agents like tulathromycin, either of the two ratios C_max_/MIC or AUC/MIC, out of all the PK/PD indices that have been researched, have shown an association with clinical outcomes [5]. In the present study, the PK/PD parameters that were better indicators of the efficacy of tulathromycin were C_max_/MIC and AUC/MIC. The concentrations of tulathromycin in serum at different time intervals empirically support that tulathromycin’s effect on *H*. *parasuis* is concentration-dependent, thus indicating that C_max_ is a suitable parameter to evaluate the PKs of a drug in killing bacteria [5]. Bactericidal activity significantly increased when concentration of tulathromycin added in blank serum increased as indicated by approximately up to 2 times the MIC in serum or 4 times the MIC in CAMHB in this study, which showed a high eradication rate of the infection. These results suggest that the concentration of tulathromycin when above the MIC is important for tulathromycin efficacy. However, because we observed that PAE of tulathromycin increased with *in vitro* time of exposure and concentration of the antibiotic, evaluation of tulathromycin efficacy by C_max_/MIC would be affected. Therefore, we, as well as other researchers, conclude that AUC/MIC is the better predictor of tulathromycin efficacy [34–36].

The measure of MPC represents the lowest drug concentration that can prevent the growth of first-step resistant mutants, and is the highest concentration within the mutant selection window (MSW), whose lowest concentration is the MIC. The ratio of AUC_0-24_/MPC is known to be an indicator of selection of antimicrobial resistance [42–46]. An an *in vitro* study of marbofloxacin determined that an AUC_0-24_/MPC > 25 h could prevent resistance selection in pigs with *H*. *parasuis* infection [42]. In the present study, the MPC against *H*. *parasuis* (13R) was 2.048 μg/mL (*in vitro*), four times higher than the MIC (0.5μg/mL, *in vitro*), and the AUC_0-24h_/MPC_CAMHB_ was 5.4 ± 1.2 h. Therefore, the use of AUC/MPC may be an advantage in maximizing efficacy and reducing the incidence of drug resistance than the use of AUC/MIC. Although we knew that tulathromycin can have a strong effect on serum, however, because of we did not obtain the MPC in serum, thus, AUC/MPC was not used for the prediction of dose regimen in this study.

Integration of PK/PD for dose optimization of tulathromycin treatment via an *in vitro* model has been reported by some researchers for target animals. In the present study, 7.2 or 8.0 mg/kg were our dose estimates to achieve the virtual eradication of *H*. *parasuis* over a ten-day period in guinea pig. Furthermore, we applied a dose conversion coefficient from guinea pig to pig [47] of 0.296 to our estimates to obtain equivalent doses of 2.1 mg/kg or 2.4 mg/kg for the target animal, pig. Our result is lower than the previous studies, 13.25 mg/kg against *Pasteurella multocida* [35] and 3.56 mg/kg against *Streptococcus suis* [34], which were higher than the recommended dose (2.5 mg/kg). In contrast, results in this study is similar to report of an estimated dosage of 2.52 mg/kg of tulathromycin against *Pasteurella multocida* [36]. The difference may be explained by the variable responses of different bacteria to tulathromycin which likely results in different PK/PD models. Our study supports the current marketed dose of tulathromycin as an appropriate dose and that the concentration of tulathromycin achieved in serum with this dosage is biologically and clinically relevant [36].

The main limitation in this study was that we did not take into account that tulathromycin specifically targets lung tissue [25, 27, 29]. In addition, differences between *in vivo* and *in vitro* conditions were not considered, for example, in *in vitro* conditions, the concentration of antibacterial decline was invariable, and because bacteria is continuously exposed to fixed concentration of antibiotics for a defined period of time, this ignores the natural rates of body clearance in drug metabolism of the animal [48]. Thus, the estimated dose regimen should be validated in swine in a future study.

## Conclusions

The present study characterized the *in vitro* efficacy of tulathromycin against *H*. *parasuis* in a neutropenic infected guinea pig model. The *in vitro* data showed that tulathromycin exerted concentration-dependent bactericidal activity against *H*. *parasuis*, had a greater effect in serum than in CAMHB, and had long PAE. The data from this study can help improve tulathromycin efficacy with respect to bacteriological and clinical outcomes by providing a rational approach to the design of optimal dosage regimens (2.1–2.4 mg/kg) for target animals.
